# Nutritional Evaluation and Risk Assessment of the Exposure to Essential and Toxic Elements in Dogs and Cats through the Consumption of Pelleted Dry Food: How Important Is the Quality of the Feed?

**DOI:** 10.3390/toxics9060133

**Published:** 2021-06-05

**Authors:** Ana Macías-Montes, Manuel Zumbado, Octavio P. Luzardo, Ángel Rodríguez-Hernández, Andrea Acosta-Dacal, Cristian Rial-Berriel, Luis D. Boada, Luis Alberto Henríquez-Hernández

**Affiliations:** 1 Toxicology Unit, Research Institute of Biomedical and Health Sciences (IUIBS), Universidad de Las Palmas de Gran Canaria, Paseo Blas Cabrera Felipe s/n, 35016 Las Palmas, Spain; ana.macias@ulpgc.es (A.M.-M.); manuel.zumbado@ulpgc.es (M.Z.); octavio.perez@ulpgc.es (O.P.L.); anrodrivet@gmail.com (Á.R.-H.); andrea.acosta@ulpgc.es (A.A.-D.); cristian.rial@ulpgc.es (C.R.-B.); luis.boada@ulpgc.es (L.D.B.); 2 Spanish Biomedical Research Centre in Physiopathology of Obesity and Nutrition (CIBERObn), Paseo Blas Cabrera Felipe s/n, 35016 Las Palmas, Spain

**Keywords:** risk assessment, toxic elements, heavy metals, food safety, animal feed

## Abstract

Dry feed for pets lacks specific legislation regarding maximum residue limits for inorganic elements. The aim of the present study was to determine the content of 43 inorganic elements in dog and cat feed, studying whether there were differences according to the supposed quality of the food and performing the risk assessment for health. Thirty-one and thirty packages of pelleted dry food for cats and dogs, respectively, were analyzed. After acidic microwave-assisted digestion, elements were detected and quantified by Inductively Coupled Plasma-Mass Spectrometry (ICP-MS). In general, we did not observe important differences in the content of elements according to the supposed quality of the brand. Among trace elements, selenium and manganese are above the dietary reference value. Arsenic and mercury showed the highest acute hazard indexes, which make them risk factors for the health of dogs and cats. Aluminum, uranium, antimony and vanadium contents were above the toxic reference value and showed the highest acute hazard indexes. It is necessary to improve the legislation regarding the food safety of pets, for their health and to protect the rights of consumers.

## 1. Introduction

According to recent data, the European pet population in 2019 was nearly 300 million, with the cat and dog population being two-thirds of the total [1]. The estimated number of European Union (EU) households owning at least one pet animal is 85 million. In Spain, there are registered almost 7 million dogs and 4 million cats [2], numbers that are probably higher in the case of cats due to the low ratio of registered animals. In the last 5 years, the number of pets in Spain has increased by 40%. At present, the census of dogs exceeds that of babies, children and adolescents under 15 years of age [2,3]. This increase translates into higher expenses for family units for feeding and caring for domestic animals. The pet sector commands EUR 278 million in Spain only, for medicine and food [4].

In the EU, the annual growth rate of the pet food industry has increased 2.6% over the past 3 years, with the annual value of pet-related products and services being €19.7 billion [1]. According to The European Pet Food Industry, the number of pet food producing companies is 132 and the annual sales of pet food products increased to 8.5 million tons in 2019 (turnover: EUR 21 billion) [1]. Of this amount, dry food accounted for 84.4% and 70.4% of dog and cat food, respectively [5]. The III Edition of the Annual Study on Pets in Spain reveals an average expenditure on pet food of EUR 69.98 per month [6]. The same study maintains that quality is the most important factor to take into account when deciding on one brand over another. However, there are no objective criteria applied to the pet food industry which must be met in order to obtain a quality certificate for the food. In general terms, and according to specialized studies, the price of the product seems to be the main variable that a consumer has to take into account when deciding on a quality product, in such a way that a more expensive product is perceived by the consumer as of better quality [7]. In other words, the quality of the product is absolutely subjective, conditioned by the reputation of the brand, the advertising and sales strategies. With an expanding business volume and wide profit margins, it is necessary to clearly differentiate the quality of pet food in order to improve the food safety of animals and protect the consumer, who is, in the end, the owner of each pet.

The European legislation on undesirable substances in animal feed regulates the maximum content of these kind of substances, including toxic elements such as arsenic, lead, mercury and cadmium [8]. The European regulation aims to harmonise the conditions for the market placement and use of feed, in order to ensure a high level of feed safety and, thus, a high level of protection of public health [9]. Despite the existence of specific legislation, the control seems to be focused on the feeding of production animals rather than on pets. As an example of this lack of control, recent studies have reported high levels of mycotoxins [10,11] and chlorinated pollutants [12] in pet food. Both groups of pollutants are legislated but, apparently, not controlled [8].

Although there is white label pet food rated as quality by the Spanish Organization of Consumers and Users (OCU) [13], 92% of pet owners opt for brand-name feed, making “quality” the most important factor when deciding on one brand over another [6]. Because the quality of pet food does not meet controlled standards, marketers commonly label pet food as “premium” or “ultra-premium”. With this strategy, consumers are led to believe that they are purchasing higher quality food, both nutritionally and from a food safety point of view. In the consumer’s mind, this would imply that the food in question should have a lower level of contamination of harmful chemicals. However, this assumption is not supported by published scientific data that have shown that there are no differences in mycotoxin levels depending on whether the feed is “of quality” or not [10]. In some cases, the mineral composition of dry dog foods does not conform to the requirements given by nutritional guidelines, exceeding the legal limit for iron or zinc in a large number of samples [14].

Since 1976, when the first article about heavy metal levels in pet food was published [15], barely a dozen articles have been published, with most of them focused on heavy metals and metalloids [16,17,18,19,20,21,22,23]. Although the toxic elements analyzed are usually below the permitted limits, cases have been reported in which the levels of arsenic and heavy metals consumed are higher in pets [19]. Only a few studies have performed a health risk assessment, probably due to the low interest in pet food safety compared to that of production animals, which are part of our food chain and our own food safety. Recent studies have been published showing that, for example, arsenic contained in rice-based diets poses a health risk to dogs [16]. Similar results have been published regarding mercury [18]. In any case, the body of knowledge is mainly focused on about ten elements, including the essential elements, the main heavy metals (lead, mercury and cadmium), and arsenic which, in addition, are the legislated elements [8,9]. However, according to studies previously published by our group, we know that the number of potentially hazardous inorganic elements that reach living beings through the diet is very large [24].

The present study aims to carry out an exhaustive analysis of forty-three elements—including essential elements, toxic elements and potentially toxic elements, rare earth elements and other minor elements—in a total of thirty-one packages of pelleted dry food for cats and thirty packages of pelleted dry food for dogs, comparing brands of supposed quality with those that are not, evaluating the quality of the food in relation to the contribution of essential elements and carrying out an estimate of the health risk.

In general, no differences were found according to the reputation of the brands. Arsenic, cadmium, aluminum, barium, molybdenum, nickel, antimony, strontium, thallium and vanadium reach, at least, 50% of the reference toxic value for cat and dog food samples.

## 2. Materials and Methods

### 2.1. Sampling

Thirty-one packages of pelleted dry food for cats (0.4–2 kg) and thirty packages of pelleted dry food for dogs (1.25–15 kg) were purchased. Each brand was purchased in duplicate in shops located in Gran Canaria (Canary Islands, Spain) from specialized stores, retail outlets and supermarkets. All samples had an expiration date of more than 4 months from the date of purchase.

Different price brands were selected to cover the entire price range present in the market. The average price, in EUR/kg, was calculated for each sample taking into account that the smaller the package, the more expensive the product. The supposed quality of the brand was associated with the price of the product. The median price was the value established to separate the samples into two different groups: high–mid cost vs. low cost. Thus, a total of 12 brands of dog food were high–mid cost while 18 brands were low cost. Regarding cat food, 14 brands were high–mid cost while 17 were low cost. The cost for dog food brands ranged from 0.9 to 9.0 €/kg while the cost for cat food brands ranged from 0.65 to 13.5 €/kg selected. 

The present study did not include samples of bulk feed.

Since feed flavor can be associated with the presence of certain types of contaminants, feeds with different protein origins were selected, including fish (salmon and tuna) and meat (chicken, beef, pork and turkey).

All the samples had national and/or international distribution, but none of the foods were manufactured in Gran Canaria. The samples were stored in a dark and dry environment at room temperature without removing them from the commercial packaging until analysis.

The sampling method was similar to that used previously by our group [10].

### 2.2. Standards and Elements

A total of 43 elements were analyzed, including essential elements, elements contained in the priority list of the Agency for Toxic Substances and Disease Registry (ATSDR), and rare earth elements (REEs) and other minority elements (MEs). Thus, a wide spectrum of substances was covered, ranging from essential elements in the diet to emerging pollutants—of concern because of their massive employment in the manufacturing of electric devices [25]—including heavy metals and metalloids classically considered as toxic. The complete list of elements is as follows (Supplementary Materials, Table S1): copper, iron, manganese, selenium and zinc (essential elements); arsenic, cadmium, mercury and lead (toxic elements); silver, aluminum, barium, beryllium, molybdenum, nickel, antimony, tin, strontium, thallium, uranium and vanadium (potentially toxic elements); cerium, dysprosium, erbium, europium, gallium, gadolinium, holmium, indium, lanthanum, lutetium, niobium, neodymium, osmium, praseodymium, platinum, ruthenium, samarium, tantalum, terbium, thulium, yttrium and ytterbium (RREs and MEs).

Pure standards of elements in 5% HNO_3_ solution were purchased from CPA Chem (Stara Zagora, Bulgaria). Two standard curves were made as previously reported [24]: one using a commercial multi-element mixture containing essential elements and toxic elements and the other multi-element mixture made ad hoc in our laboratory containing the RREs and MEs. Each curve had 12 points and a range of 0–100 ng/mL.

### 2.3. Analytical Procedure

The samples were thoroughly mixed in their original package before proceeding. Subsequently, the duplicate samples were mixed equally to form a single 30-g analysis subsample. Each sample was manually homogenized in a mortar. Then, a total of 500 mg were acid-digested in a microwave digester (Ethos Up, Milestone SRL, Sorisole, Italy).

The internal standard solution was composed of scandium, germanium, rhodium and iridium, each one at a stock concentration of 20 mg/mL. A total of 50 µL of the internal standard solution, 2.5 mL of nitric acid (65%) and 7.5 mL of Milli-Q water were added to each sample.

All samples were then digested as follows: Step 1: a power (W), temperature (C), and time (min) of 1800, 100, and 5, respectively; Step 2: 1800, 150, and 5; Step 3: 1800, 200, and 8; Step 4: 1800, 200, and 7, as previously reported [24]. The digested samples were quantitatively transferred into conic bottom polypropylene tubes and quantitatively diluted up to 15 mL with Mili-Q water. Three samples were taken from each digestion vessel to obtain a triplicate measurement of each sample. A reagent blank, prepared as the samples, was included every 14 samples in the analytical batch.

An Agilent 7900 ICP-MS (Agilent Technologies, Tokyo (Japan)) was employed for all measurements. All the data were acquired and processed with Agilent MassHunter Data Analysis software (version 4.2, Agilent Technologies, Palo Alto, CA, USA). 

The entire/complete procedure was validated prior to its use in the analyses of samples [24]. All determinations were performed in triplicate from each vial. Recoveries obtained ranged from 87 to 118% for toxic and essential elements. Linear calibration curves were found for all elements (regression coefficients ≥ 0.998).

Limits of detection (LODs) and quantification (LOQs) were calculated as the concentration of the element that produced a signal that was three and ten times higher than that of the averaged blanks, respectively. The sample LOQs were calculated by multiplying the instrumental LOQ by the dilution factor (1:10 *v*:*v*). LODs and LOQs are included in Supplementary Materials, Table S1.

### 2.4. Estimation of Dietary Intake, Nutritional and Health Risk Assessment

A quantitative exposure assessment was performed to try to estimate the probability and severity of adverse health effects caused by inorganic elements, in cats and dogs. It has to be highlighted that, although for some elements the nutritional needs and potentially toxic doses are established, for most of the elements considered in the present study there are no toxicological reference values for dogs and cats. In the absence of appropriate reference values for pets, we used the same reference points as those for humans assuming that, although exceptions exist, dogs and cats are not substantially different from humans [10]. This extrapolation means that the results should be interpreted with caution.

The dietary reference value (DRV) [26], the tolerable upper daily intake level—as the maximum level of total chronic intake—[27] and the total reference value (TRV)—as the dose established for a given chemical and a route-specific capable of critical health effects over time—[28] were taken into account. For REEs and MEs, TRV was set at 61 μg/kg body weight, considered RREs as a sum, as previously reported [24,29].

The estimated short-term intake (ESTI) as the acute health risk was calculated as follows [10,30]:

(1) ESTI=HRE×K

where *HRE* represents the highest residue level of elements found in the series and *K* is the recommended amount of feed per kilo and day of that feed, according to the manufacturer’s recommendation. *ESTI* is measured in ng of element per kilogram of body mass per day.

The acute hazard index (aHI), as the ratio between the exposure to a single dose of a toxic substance and the acute reference dose of toxicity for it, was calculated as follows [10]:

(2) $$aHI=\frac{ESTI}{ARfD}$$

where *ARfD* represents the Acute Reference Dose, defined as an estimation of the amount of a substance in food or drinking water, normally expressed on a body mass basis, that can be ingested in a period of 24 h or less without appreciable health risks to the consumer on the basis of all known facts at the time of the evaluation and obtained for each element from the Integrated Risk Information System [28].

### 2.5. Statistical Analysis

Descriptive analyses, including mean, standard deviation, median, range and proportions, were calculated for all variables. A random value between LOQ and LOD was automatically assigned to those data between both data, as previously reported [24,31,32]. Those data below LOD were considered as non-detected. Since most of the data series did not follow a normal distribution*,* non-parametric tests were used.

PASW Statistics v 25.0 (SPSS Inc., Chicago, IL, USA) was used to manage the database and to perform the statistical analyses. Probability levels of <0.05 (two-tailed) were considered statistically significant.

## 3. Results and Discussion

### 3.1. Content of Elements in Pelleted Dry Food for Dogs and Cats

Descriptive analyses of elements—including essential elements, toxic elements and potentially toxic elements—in pelleted dry food for dogs and cats are shown in Table 1 and Table 2. Results were divided according to the type of brand: premium vs. low-cost brands, according to the mean price of each one (EUR 5.20 vs. EUR 2.53, *p* value < 0.01; and EUR 8.73 vs. EUR 2.72, *p* value < 0.001; for dry food for dogs and cats, respectively). Following this strategy, we investigated if the assumed quality of the product was related to different inputs of essential elements or different levels of harmful substances.

Median values of iron were significantly higher among low-cost brands of food for dogs and cats (*p* value = 0.0037 and *p* < 0.0001, respectively; Table 1). Beyond some differences between the types of dog food—referring to copper and zinc—no statistically significant differences were observed regarding the content of essential elements between both types of food: premium brands vs. low-cost brands. The brands of a supposed quality show on the label that they have been supplemented with essential elements, among which are usually copper, iron, manganese, selenium and zinc. According to our results, this supplementation does not seem to be reflected in the final amount of these elements since no significant differences were observed between low and high-quality feeds. Similar results have been observed in previous studies in which the content of the analyzed elements was equal regardless of the type of food [33]. Although there is specific European legislation on undesirable substances in animal feed [8] it seems necessary to increase the controls to ensure that the legislation is complied with [34], especially when most of the efforts are focused on controlling the feed of production animals, which influence people’s health [9].

Regardless of price and quality, all pet food must be safe. However, in the consumer’s mind, a more expensive food must be nutritionally better and healthier than a cheaper one. This implies, among many things, lower levels of pollutants. This assumption would be achieved by using higher quality raw materials—more expensive, by definition—and increasing controls to comply with legislation. According to our results, lead, aluminum, antimony, tin and strontium content were higher among low-cost brands of pelleted dry food for dogs (5 out of 16 toxic and potentially toxic elements considered in the study, excluding RREs and MEs). The content of aluminum, barium and strontium were higher among low-cost brands of pelleted dry food for cats (3 out of 16 toxic and potentially toxic elements considered in the study). However, despite these results, no significant differences in the amount of most of the toxic and potentially toxic elements were observed between premium and low-cost brands, neither for pelleted dry food for dogs nor for cats (Table 2). No differences in the content of REEs and MEs, considered as a sum of 19 different elements, were found. Although, with nuances, these results suggest that a more expensive feed does not necessarily imply that it is of higher quality in terms of inorganic contamination.

In view of these results, it appears that feed price does not determine the contaminant profile. However, the composition does seem to have an influence. Therefore, we investigated whether the contaminant profile was different between fish-based and meat-based feeds. This comparison could only be made in cat feed since only two dog feed samples in the series analyzed were fish-based. Median values of arsenic, cadmium and mercury were significantly higher among fish-based cat foods: 191.1 ng/g vs. 89.9 ng/g (*p* = 0.009), 49.1 ng/g vs. 38.2 ng/g (*p* = 0.015), and 5.4 ng/g vs. 1.7 ng/g (*p* = 0.0003), respectively. The raw material that forms the basis of the feed has an important influence on the contamination profile, as previously observed [16].

Although labeling of the analytical constituents of compound feed for non-food producing animals is legislated [9], it refers to calcium, sodium, phosphorus and other relevant minerals, without further specification. Other potentially toxic elements are not legislated [20].

### 3.2. Dietary Intake and Risk Assessment

Dietary reference values (DRVs) and toxic reference values (TRVs) were expressed as a percentage since the differences in absolute data between the different elements can be very wide and, therefore, more difficult to represent graphically. Details are given in Supplementary Materials, Table S1.

#### 3.2.1. Essential Elements

To determine if the food meets the nutritional need of dogs and cats, we have calculated the daily exposure to essential elements. An evaluation of the acute health risk posed by these elements was also estimated, for premium and low-cost brands.

**Figure 1:**
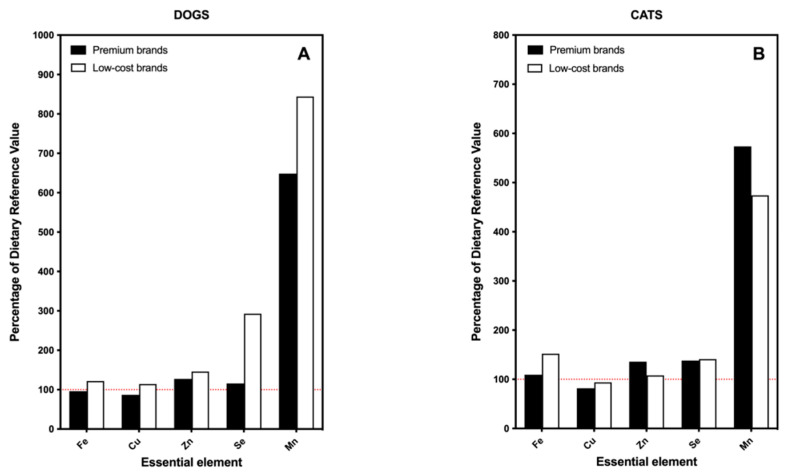
Bar plot indicating the percentage of the DRV of essential elements provided by the consumption of commercial dry food separated by the type of brand: premium vs. low-cost brands. (**A**) Results for dogs; (**B**) results for cats. Red dotted line indicates 100% of the DRV for each element.

In general terms, both types of food reached 100% of the DRV for iron, copper, selenium and zinc (Figure 1A,B). However, the selenium intake from low-cost brands of dog food accounted for almost 300% of the required nutritional requirements. The intake of manganese exceeded the DRV for both types of food brands for dogs and cats, reaching values of more than 4 times higher than the established needs and even doubling that value (8×) in the case of low-cost feed for dogs (Figure 1A). In no case did these values pose a risk of toxicity for the animals fed with the analyzed feed. For selenium, dogs seem to tolerate more than twenty times the DRV [35]. For manganese, there are very few studies on the toxicity of this element in dogs, so nothing can be hypothesized about it [36]. However, the profile of essential elements draws attention, since supplementation of feed with trace elements is usually an advertisement from premium brands and this message does not seem to correspond with the findings obtained after the analyzes. Not only are there no significant differences in the content of essential elements between premium and low-cost brands but, on some occasions, the content of these elements is not adjusted to the nutritional requirements of pets.

Recently, a published study has drawn attention to the need for routine analysis of the essential composition of raw materials before introducing any type of supplementation [14]. In this and other studies, it is common to see that the amounts of some essential elements, selenium and manganese among them, are usually over-supplemented, sometimes exceeding the established legal limits [36,37]. In that sense, our results agree with others already published, highlighting the existing discrepancies between what the pet industry says that a food provides and what it really has in terms of essential elements. The fact that only a minority of brands comply with all nutrient content claims listed on their labels [38,39] indicates that the need for legislation in this regard is urgent. Apart from not complying with the legislation related to labels and being, at times, misleading advertising, in certain circumstances the health of the pets themselves can even be put at risk.

#### 3.2.2. Toxic Elements

**Figure 2:**
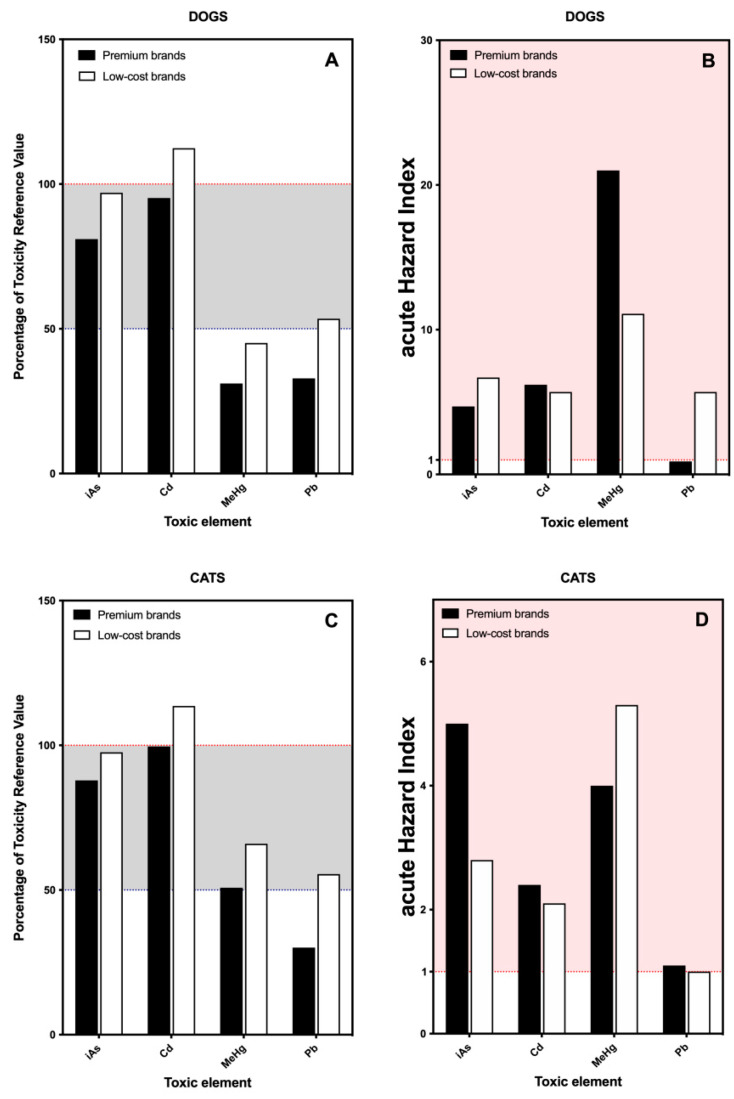
Bar plot indicating the percentage of the toxic reference value (TRV) and the acute Hazard Index (aHI) of toxic elements—arsenic (As), cadmium (Cd), mercury (Hg) and lead (Pb)—provided by the consumption of commercial dry food separated by the type of brand: premium vs. low-cost brands. (**A**,**B**) Results for dogs; (**C**,**D**) results for cats. Red dotted line indicates 100% of the TRV or the threshold for toxic effect for each element.

In the present section, we have calculated the daily exposure of dogs and cats to toxic elements to determine if the food is an important source for the intake of these substances. Evaluation of acute health risk posed by these elements was also estimated (Figure 2).

Arsenic, cadmium, mercury and lead are considered toxic elements due to their damaging capacity to living beings. For this reason, the levels of residues of these elements are strictly legislated [8]. Certain foods are usually associated with specific elements, with more or less significance. This is the case with rice and arsenic [40] or mercury and fish [41]. Our results have shown that arsenic and cadmium were close to 100% of the TRV while mercury and lead reached 50% of the TRV. Recently it has been found that very little methylmercury persisted in pet foods [42]; therefore, the mercury results refer to total mercury. Interpretation of these results should be made with caution. The results were similar for pelleted dried food for dogs and cats (Figure 2A,C). No important differences between premium and low-cost brands were found. Given that these differences are only qualitative, the interpretation of these results should be made with caution.

Toxicity is neither a binary nor an absolute term. Thus, although toxicity follows a dose-response relationship, additional parameters are needed to know “How toxic is toxic?”. The aHI establishes a relationship between the amount of toxic intake in the diet and the risk of acute toxicity, giving an approximated idea of the estimated risk that food poses to the health of the individual [30]. With the exception of lead, the toxic elements considered had an aHI higher than the threshold (Figure 2B,D) and, in cases such as mercury in premium brands for dogs, it was up to 20 times higher (Figure 2B). The presence of lead in pet feed has been historically analyzed [15,21]. Lead levels have decreased in the last 5 decades, mainly due to the environmental regulations regarding the use of lead (7.6 μg/g in 1976 [15] vs. 0.21 μg/g in the present study (Table 2)). Similarly, although cadmium has been detected in pet food, it does not appear to pose a relevant health risk [19].

Regarding arsenic and mercury, both elements are closely related to the intake of certain foods, mainly rice and fish-based products. In the present study, arsenic and mercury showed the highest aHIs, especially for cat food, regardless of the supposed quality of the feed (Figure 2B,D). A recent publication reported that cat food showed a higher content of cadmium, chromium, lead and tin than dog food [20], a finding which agrees with our results in the sense that cat food appears to have higher levels of toxic inorganic elements than dog food. The fact that cat food seems to be more contaminated could be possible due to the greater presence of derivatives from fishing and fish oils—commonly used as an elemental part of cat food—which has higher levels of contaminants such as arsenic, cadmium or mercury. Organic mercury compounds bioaccumulate in organisms and have disproportionate nervous side effects: ataxia, abnormal movements, uncontrolled howling, changes to visual, cognitive, and emotional functions, and death [43]. It has to be taken into account that cats are particularly sensitive to low doses of organic mercury [43,44] and, according to our results, aHI for mercury in cat food is 4–5 times higher than the safe level (Figure 2D). The presence of mercury in pet food has been previously reported [18,19,20,43] and, in that sense, our results coincide with the bibliography, which also insists on the control of this type of contaminations in terms of quantification and inclusion on the label.

Arsenic and mercury are usually associated with the consumption of fish/seafood. In the present study, arsenic and mercury levels were significantly higher in fish-based feeds, as shown above (Section 3.1). Thus, we explored if there was any correlation between both elements and if the correlation was the same for cat feeds—with a greater presence of this type of food—and for dog feeds (Supplementary Materials, Figure S1). This association has been previously reported with similar results to that obtained in the present study [37]. Both associations were statistically significant (*p* < 0.0001) but the Spearman’s correlation coefficient was stronger for cat feed: 0.8066 vs. 0.6641, respectively. This result suggests that cat feeds, due to their composition, have a higher combined risk of arsenic and mercury. The fact that cats are especially sensitive to organic mercury compounds makes it necessary to include information regarding contaminant residues on the label of pet food.

#### 3.2.3. Potentially Toxic Elements

**Figure 3:**
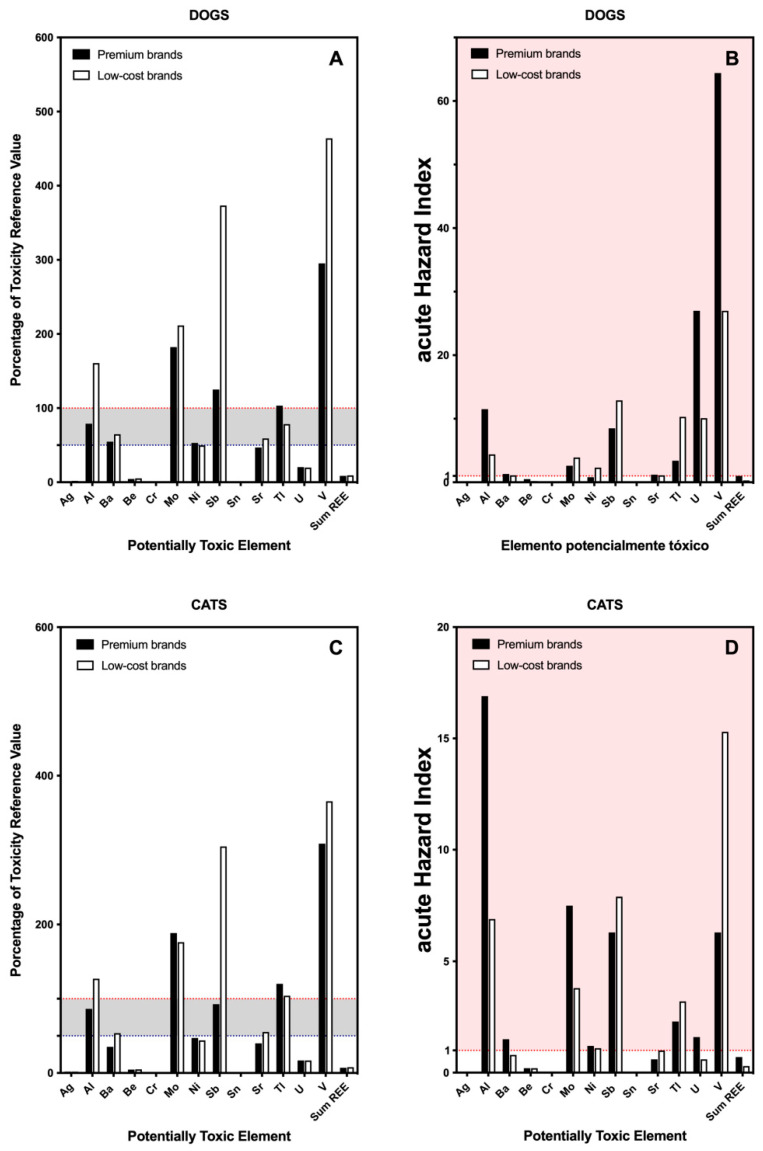
Bar plot indicating the percentage of the toxic reference value (TRV) and the acute Hazard Index (aHI) of potentially toxic elements and RREs, provided by the consumption of commercial dry food separated by the type of brand: premium vs. low-cost brands. (**A**,**B**) Results for dogs; (**C**,**D**) results for cats. Red dotted line indicates 100% of the TRV or the threshold for toxic effect, for each element. Abbreviations: Ag, silver; Al, aluminum; Ba, barium; Be, beryllium; Cr, chromium; Mo, molybdenum; Ni, nickel; Sb, antimony; Sn, tin; Sr, strontium; Tl, thallium; U, uranium; V, vanadium; Sum RRE, sum of cerium, dysprosium, erbium, europium, gallium, gadolinium, holmium, indium, lanthanide, lutetium, niobium, neodymium, praseodymium, samarium, tantalum, terbium, thulium, yttrium and ytterbium.

In the present section, we have calculated the daily exposure of dogs and cats to potentially toxic elements to determine if the food is an important source for the intake of these substances. A total of 13 elements and 19 RREs—considered as a sum—were included in this section (Supplementary Materials, Table S1). The evaluation of the acute health risk posed by these elements was also estimated (Figure 3).

Aluminum, molybdenum, antimony and vanadium showed percentages of TRVs higher than the safety values for dog and cat food, independent of the quality of the food; although low-costs brands showed, in general terms, higher TRVs than premium brands (Figure 3A,C).

To our knowledge, this is the first time that many of these potentially toxic elements have been analyzed in commercial pet food [14,22]. A previous publication has considered these elements but in home-prepared diets for adult pets [23]. Although the comparison cannot be made directly, it has caught our attention that elements such as vanadium were above the maximum tolerable levels in almost 90% of dog and cat food samples [23]. Something similar was found in the case of uranium. According to our results, although uranium did not reach values near to the TRV, the aHI was the second highest—after vanadium—reaching values 30 times higher than desirable in the case of premium brands for dogs (Figure 3B). The percentage of home-prepared diets for adult pets with uranium content above the maximum tolerable level was around 100% [23]. A similar profile was reported in a series of dry dog foods in Brazil [22]. In that study, aluminum, antimony, and uranium were detected above the maximum levels established for humans, a result that coincides with what was observed in our study, not only for dog food but also for cat food (Figure 3D). These similarities and differences between studies are attributable to the ingredients and processes used in food manufacturing, as previously suggested [19,45]. Thus, the profile of contaminants found in fish-based foods is different from that observed in foods that use red meat or other protein sources from mammals (i.e., lamb or chicken).

Aluminum is the most abundant metal in the earth’s crust but is toxic and dangerous because it does not occur naturally inside our bodies. Although air, water and soil are important, the main source of exposure is food [46]. Exposure to aluminum is related to allergies and neurological diseases but its mechanism of action is not fully understood. Some pathways of aluminum toxicity include altered enzyme activity, significantly decreasing the functionality of the affected enzymes. Different studies have reported serious health problems in animals after oral exposure to aluminum, including systemic acute toxicity, immunological, reproductive and neurologic diseases, and even death [46]. Similarly, vanadium is widely distributed in the earth’s crust (mean concentration ≈ 100 mg/kg) and it reaches living organisms mainly through food [47]. Exposure to high levels of vanadium has been associated with decreases in the number of red blood cells, increased blood pressure, neurological effects and developmental effects in animals [47]. Both aluminum and vanadium are dangerous elements for health and, in the present study, they are the elements with the highest acute hazard index, for cats and dogs, respectively. Other elements such as uranium, antimony or molybdenum have to be taken into account and may be responsible for adverse health effects on pets.

In view of these observations, it is necessary to increase the control of pet food in order to comply with current legislation [8,9].

## 4. Conclusions

In general, we did not observe important differences in the content of elements according to the supposed quality of the analyzed feed; therefore, premium brands do not represent any clear advantage over other lower-priced ones. Among trace elements, selenium and manganese are above the dietary reference value, although they do not reach toxic levels. Among the toxic elements, arsenic and mercury showed the highest acute hazard indexes, which make them risk factors for the health of dogs and cats. Among potentially toxic elements, the content of aluminum and vanadium were above the toxic reference value. Together with uranium and antimony, they showed the highest acute hazard indexes. The combined effect of these elements may be greater than the one they have individually. It is necessary to improve the controls in order to comply with current legislation.

## Figures and Tables

**Table 1:** Content of essential elements in pelleted dry food for dogs and cats, separated by commercial quality (based on price). Results are expressed in μg_element_/g_feed_.

	Premium Brands (*n* = 12) ^1^			Low-Cost Brands (*n* = 18) ^2^			
DOG FEED	Mean ± SD	Median	p25–p75	Mean ± SD	Median	p25–p75	*p*
Fe	122.0 ± 22.4	122.0	104.8–141.3	176.5 ± 64.7	167.7	138.7–204.1	0.0037
Cu	13.4 ± 2.3	13.8	11.5–14.7	14.9 ± 1.6	14.9	14.4–15.9	0.0252
Zn	174.8 ± 25.2	181.6	157.6–193.0	145.5 ± 23.6	139.2	132.7–154.9	0.0037
Se (ng/g)	660.5 ± 299.4	604.8	494.8–751.4	710.4 ± 205.9	648.4	572.7–803.3	n.s.
Mn	45.9 ± 19.1	51.3	26.3–64.3	46.6 ± 14.0	43.8	39.9–58.6	n.s.
	**Premium Brands (*n* = 14) ^3^**			**Low-Cost Brands (*n* = 17) ^4^**			
**CAT FEED**	**Mean ± SD**	**Median**	**p25–p75**	**Mean ± SD**	**Median**	**p25–p75**	***p***
Fe	130.9 ± 24.2	138.9	115.1–143.6	189.7 ± 43.4	193.4	169.2–211.8	< 0.0001
Cu	13.4 ± 3.2	13.2	10.0–15.0	14.9 ± 3.9	14.9	13.2–15.7	n.s.
Zn	177.3 ± 30.1	192.2	147.3–197.6	160.8 ± 43.8	153.9	128.4–196.3	n.s.
Se	761.2 ± 154.4	722.2	650.1–834.1	775.2 ± 187.3	738.2	659.1–888.1	n.s.
Mn	45.1 ± 24.4	48.8	17.6–67.9	42.2 ± 15.7	37.5	30.6–55.9	n.s.

Abbreviations: SD, standard deviation; p25–p75, percentiles 25th and 75th of the distribution; n.s., non-significant. ^1^ Mean price of premium brands for dogs: EUR 5.20. ^2^ Mean price of low-cost brands for dogs: EUR 2.53. ^3^ Mean price of premium brands for cats: EUR 8.73.^4^ Mean price of low-cost brands for cats: EUR 2.72.

**Table 2:** Content of toxic and potentially toxic elements in pelleted dry food for dogs and cats, separated by commercial quality (based on price). Results are expressed in ng_element_/g_feed_.

DOF FEED	Premium Brands ^1^			Low-Cost Brands ^2^			
Toxic Element	Mean ± SD	Median	p25–p75	Mean ± SD	Median	p25–p75	*p*
As (total)	159.3 ± 179.8	101.3	51.2–160.4	180.2 ± 192.3	121.3	88.3–176.3	n.s.
Cd	76.3 ± 73.2	39.7	34.6–86.4	96.1 ± 79.0	46.8	38.5–171.1	n.s.
Hg (total)	10.2 ± 29.76	1.6	0.9–2.9	6.6 ± 14.5	2.3	1.6–4.5	n.s.
Pb	91.6 ± 50.7	82.2	58.8–115.3	250.1 ± 347.5	133.7	99.1–215.4	0.0108
**Potentially Toxic Element**	**Mean ± SD**	**Median**	**p25–p75**	**Mean ± SD**	**Median**	**p25–p75**	***p***
Ag	4.9 ± 6.3	3.1	2.7–4.3	5.3 ± 3.7	3.7	2.7–8.7	n.s.
Al	89,142 ± 143,107	32,869	49,034–85,000	78,489 ± 47,574	66,972	49,034–85,000	0.0252
Ba	4843 ± 2311	4555	3196–5789	5285 ± 1746	5395	3470–6282	n.s.
Be	7.9 ± 11.5	3.7	1.8–10.1	4.9 ± 4.2	4.3	2.5–5.3	n.s.
Mo	405.8 ± 86.9	379.8	328.3–506.2	455.4 ± 142.8	440.6	333.7–522.1	n.s.
Ni	481.6 ± 129.5	441.8	376.7–631.5	503.6 ± 401.8	415.2	337.4–476.8	n.s.
Sb	34.7 ± 38.8	20.9	9.8–50.1	79.8 ± 64.4	62.2	26.7–130.8	0.0176
Sn	21.3 ± 15.1	20.3	12.1–25.1	40.2 ± 29.5	35.2	15.0–43.6	0.0450
Sr	12,395 ± 6145	11,735	8659–13,396	16,214 ± 5312	14,763	11,646–17,878	0.0221
Tl	3.8 ± 2.4	3.0	2.2–5.1	4.6 ± 7.1	2.3	2.0–4.5	n.s.
U	368.7 ± 951.7	25.5	14.8–77.9	122.4 ± 322.4	24.7	15.6–35.7	n.s.
V	386.2 ± 723.6	123.0	99.5–241.2	258.5 ± 247.5	193.3	144.4–252.4	n.s.
∑REE and ME ^1^	406.9 ± 609.4	187.1	117.2–284.4	265.9–185.4	200.1	126.1–320.2	n.s.
**CAT FEED**	**Premium Brands ^3^**			**Low-Cost Brands ^4^**			
**Toxic Element**	**Mean ± SD**	**Median**	**p25–p75**	**Mean ± SD**	**Median**	**p25–p75**	***p***
As (total)	172.1 ± 151.9	109.8	66.3–215.6	147.3 ± 81.8	122.1	86.4–191.9	n.s.
Cd	43.0 ± 17.6	42.7	32.4–60.7	47.9 ± 17.6	47.4	32.4–60.7	n.s.
Hg (total)	4.6 ± 5.3	2.1	1.5–5.4	6.4 ± 7.2	2.8	1.9–10.9	n.s.
Pb	84.0 ± 59.8	75.4	60.8–84.5	130.9 ± 52.7	138.7	85.2–159.9	0.0037
**Potentially Toxic Element**	**Mean ± SD**	**Median**	**p25–p75**	**Mean ± SD**	**Median**	**p25–p75**	***p***
Ag	4.0 ± 1.9	3.5	2.6–5.5	4.1 ± 1.4	3.7	3.1–5.3	n.s.
Al	81,682 ± 172,641	36,411	30,964–46,764	72,865 ± 64,462	52,905	41,005–80,819	0.0202
Ba	3683 ± 2463	2996	2642–3638	4640 ± 1281	4465	3944–5575	0.0051
Be	4.0 ± 4.1	3.3	1.3–4.6	4.5 ± 2.9	4.1	2.8–5.2	n.s.
Mo	462.4 ± 308.1	392.5	348.3–434.6	427.0 ± 177.1	367.2	340.9–472.8	n.s.
Ni	469.0 ± 192.9	395.6	350.8–555.1	425.2 ± 174.7	365.5	332.1–459.9	n.s.
Sb	41.9 ± 40.8	23.0	11.4–67.6	56.1 ± 35.9	50.8	27.7–74.4	n.s.
Sn	31.3 ± 26.6	20.4	14.1–34.8	41.0 ± 34.1	28.2	15.8–58.9	n.s.
Sr	10,629 ± 2664	9973	8895–12,711	15,472 ± 5735	13,833	11,362–20,589	0.0137
Tl	3.7 ± 1.3	3.5	2.7–4.3	3.4 ± 1.8	3.1	2.6–3.5	n.s.
U	37.4 ± 46.4	22.4	16.6–43.1	25.1 ± 14.8	21.1	17.3–26.2	n.s.
V	144.4 ± 54.3	130.6	111.4–191.0	202.2 ± 144.1	152.3	111.6–230.0	n.s.
∑REE and ME ^1^	287.3 ± 445.0	176.7	127.3–200.0	272.4 ± 194.5	200.6	144.6–370.4	n.s.

^1^ This is the sum of the individual content of Ce, Dy, Er, Eu, Ga, Gd, Ho, In, La, Lu, Nb, Nd, Pr, Sm, Ta, Tb, Tm, Y, Yb. Abbreviations: SD, standard deviation; p25–p75, percentiles 25th and 75th of the distribution; n.s., non-significant. ^1^ Mean price of premium brands for dogs (*n* = 12): EUR 5.20. ^2^ Mean price of low-cost brands for dogs (*n* = 18): EUR 2.53. ^3^ Mean price of premium brands for cats (*n* = 14): EUR 8.73. ^4^ Mean price of low-cost brands for cats (*n* = 17): EUR 2.72.

## Data Availability

The data presented in this study are available on request from the corresponding author.

## Supplementary Materials

The following are available online at https://www.mdpi.com/article/10.3390/toxics9060133/s1, Table S1: Complete list of elements included in the study.
