# Supplementary material for: Nutritional Evaluation and Risk Assessment of the Exposure to Essential and Toxic Elements in Dogs and Cats through the Consumption of Pelleted Dry Food: How Important Is the Quality of the Feed?
Source: Toxics. 2021 Jun 5;9(6):133. doi: 10.3390/toxics9060133 (PMC8227297; doi:10.3390/toxics9060133)
Supplement: Supplementary file 1 [file toxics-09-00133-s001.zip › toxics-1214240-SI.pdf]

toxics

MDPI

Article

Supplementary Materials: Nutritional Evaluation and Risk Assessment of the Exposure to Essential and Toxic Elements in Dogs and Cats through the Consumption of Pelleted Dry Food: How Important Is the Quality of the Feed?

Ana Macías-Montes, Manuel Zumbado, Octavio P. Luzardo, Ángel Rodríguez-Hernández, Andrea Acosta-Dacal, Cristian Rial-Berriel, Luis D. Boada and Luis Alberto Henríquez-Hernández

Table S1. Complete list of elements included in the study. LOD, LOQ, DRV, TRV and maximum permitted content were included.

**Essential elements**	**Symbol**	**LOD (ng/g)**	**LOQ (ng/g)**	**DRV (ng/kg/day) (1)**	
Copper	Cu	0.4	1.7	380000	
Iron	Fe	5	24.6	3050000	
Manganese	Mn	0.1	0.4	190000	
Selenium	Se	0.05	0.2	12550	
Zinc	Zn	5	51.1	3410000	
ATSDR's Priority Pollutant list					
Toxic Elements				TRV (ng/kg/day) (2)	Maximum Permitted Content (mg/kg) (3)
Arsenic (inorganic)	iAs	0.05	0.2	300	2
Cadmium	Cd	0.02	0.1	1000	10
Mercury (methyl)	MeHg	0.02	0.2	100	0.1
Lead	Pb	0.1	0.4	6000	2
Potentially toxic elements					
Silver	Ag	0.05	0.1	5000	
Aluminium	Al	7.5	38.4	1000000	
Barium	Ba	0.2	1	200000	
Beryllium	Be	0.05	0.1	2000	
Molybdenum	Mo	0.05	0.2	5000	
Nickel	Ni	1	8	20000	
Antimony	Sb	0.05	0.1	400	
Tin	Sn	0.05	0.1	600000	
Strontium	Sr	0.1	0.5	600000	
Thallium	Tl	0.05	0.1		
Uranium	U	0.05	0.1	3000	
Vanadium	V	0.05	0.1	1000	
Rare Earth Elements and Elements Used in High Tech Devices					
Cerium	Ce	0.05	0.1		
Dysprosium	Dy	0.05	0.1		
Erbium	Er	0.05	0.1		
Europium	Eu	0.05	0.1		
Gallium	Ga	0.05	0.1		

Gadolinium	Gd	0.05	0.1	
Holmium	Ho	0.05	0.1	
Indium	In	0.05	0.1	
Lanthanum	La	0.05	0.1	
Lutetium	Lu	0.05	0.1	
Niobium	Nb	0.05	0.1	
Neodymium	Nd	0.05	0.1	
Osmium	Os	0.05	0.1	
Praseodymium	Pr	0.05	0.1	
Platinum	Pt	0.05	0.1	
Ruthenium	Ru	0.05	0.1	
Samarium	Sm	0.05	0.1	
Tantalum	Ta	0.05	0.1	
Terbium	Tb	0.05	0.1	
Thulium	Tm	0.05	0.1	
Yttrium	Y	0.05	0.1	
Ytterbium	Yb	0.05	0.1	
Sum REE				61000 (4)

(1) DRVs were obtained from National Research Council (2006) Nutrient requirements of dogs and cats. (2) TRVs were obtained at EPA - Integrated Risk Information System, available at https://www.epa.gov/iris. (3) Maximum permitted content in products intended for animal food was obtained from Directive 2002/32/EC. (4) TRV for the sum of RRE was obtained from: Sci Rep 2017, 7, 9772; and Chemosphere 2017, 168,578-582.

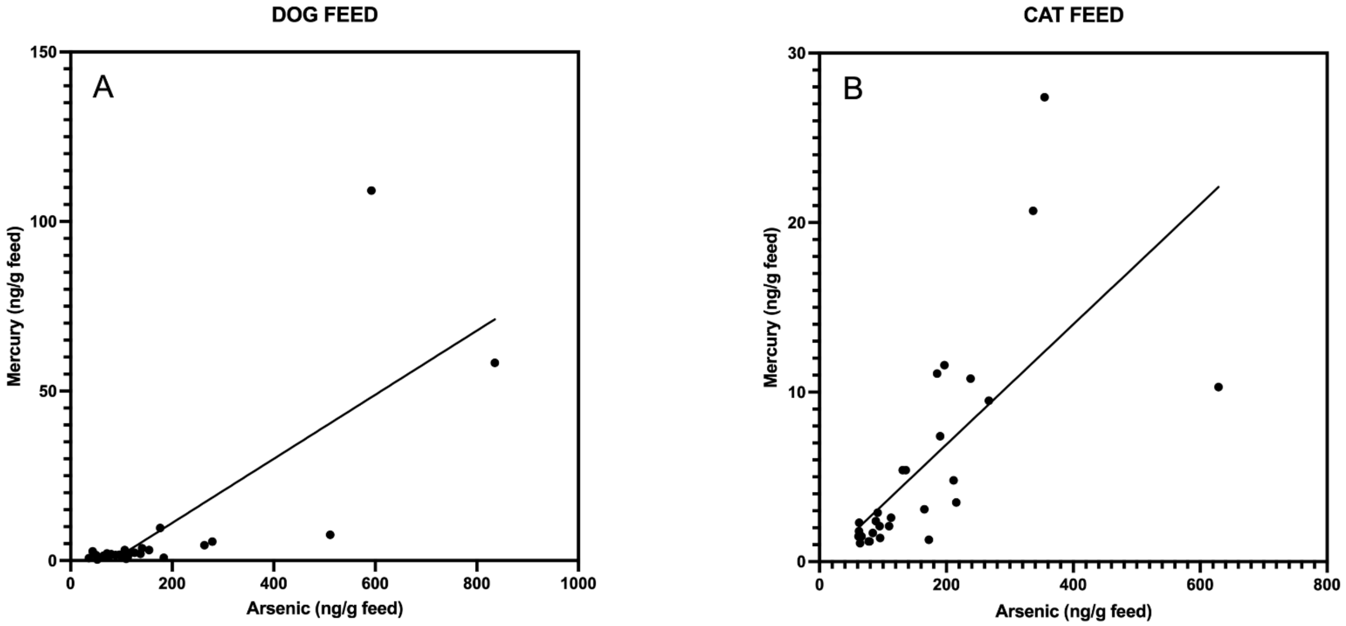

Figure S1.: Bivariate correlation between the concentration of arsenic and mercury in dog (A) and cat (B) feed.
